# Clinical Outcome in *KLHL24* Cardiomyopathy

**DOI:** 10.1161/CIRCGEN.122.003998

**Published:** 2023-05-16

**Authors:** Mathilde C.S.C. Vermeer, Karla F. Arevalo Gomez, Martijn F. Hoes, Jasper Tromp, Job A.J. Verdonschot, Michiel T.H.M. Henkens, Herman H.W. Silljé, Maria C. Bolling, Peter van der Meer

**Affiliations:** 1 Department of Cardiology (M.C.S.C.V., K.F.A.G., J.T., H.H.W.S., P.v.d.M.), Center for Blistering Diseases, University of Groningen, University Medical Center Groningen, The Netherlands.; 2 Department of Dermatology (M.C.B.), Center for Blistering Diseases, University of Groningen, University Medical Center Groningen, The Netherlands.; 3 Department of Clinical Genetics (M.F.H., J.A.J.V.), Maastricht University Medical Center, The Netherlands.; 4 Department of Cardiology (M.T.H.M.H.), Maastricht University Medical Center, The Netherlands.; 5 Department of Pathology (M.T.H.M.H.), Maastricht University Medical Center, The Netherlands.; 6 Department of Cardiology, Faculty of Health, Medicine and Life Sciences, Maastricht University, The Netherlands (M.F.H.).; 7 CARIM School for Cardiovascular Diseases, Maastricht, the Netherlands (M.F.H., M.T.H.M.H.).; 8 Saw Swee Hock School of Public Health, National University of Singapore & National University Health System (J.T.).; 9 Duke-NUS medical school Singapore (J.T.).

**Keywords:** cardiomyopathy, dilated, cardiomyopathy, hypertrophic, desmin, heart failure

Pathogenic variants in Kelch-like family member 24 (*KLHL24*; NM_017644.3) were recently identified as a new cause for skin fragility and cardiomyopathy. KLHL24 is part of a ubiquitin-ligase complex and mediates substrate recognition of intermediate filaments for proteasomal degradation (ie, keratins,^1,2^ vimentin,^2^ and desmin^3,4^). Several studies have shown that patients with heterozygous gain-of-function variants (HET-GOF), typically born with epidermolysis bullosa simplex,^1,2^ can develop dilated cardiomyopathy (DCM) with desmin-deficiency.^3^ Meanwhile, hypertrophic cardiomyopathy (HCM) with desmin-overload has been determined in patients with homozygous loss-of-function variants (HOM-LOF).^4^ This meta-analysis aims to summarize the findings of previous patient studies to determine the clinical outcome in *KLHL24* cardiomyopathy.

Observational studies (2016–2022) on KLHL24 found in PUBMED were included in this analysis. The data are available from the corresponding authors upon reasonable request. Meanwhile, an Institutional Review Board approval for this study was not required, as this is a meta-analysis. Patients were stratified according to their genotype (HOM-LOF and HET-GOF) and the study outcomes were cardiomyopathy diagnosis and cardiovascular events. Cardiovascular events were defined as sudden cardiac death, death from heart failure, or heart transplantation. Kaplan-Meier curves were constructed to visualize the age at diagnosis and cardiovascular event free-survival. For the HET-GOF group, this analysis was stratified by sex, and log-rank testing was used to test for significant differences in the distribution. To achieve between-group balance, the sex-stratified Kaplan-Meier curves were weighted for age and cohort.

In total, 73 patients from 14 studies were included in this analysis, and Figure (A) shows their geographic distribution. In general, patients had a median age (interquartile range) of 18 (7–33) years, at the time of the respected study publication. Fifty-three percent were men, 38% were diagnosed with cardiomyopathy, and 84% were patients with HET-GOF variants. Patients with HOM-LOF variants had a median age of 27 (26–31) years, and 55% were male (Figure [B]). HOM-LOF variants c.917G>A (p.[Arg306His]) and c.1048G>T (p.[Glu350*]) segregated in 2 Middle-Eastern families, reporting 11 patients born from seemingly unaffected consanguineous heterozygous parents. All patients with HOM-LOF were diagnosed with HCM before the age of 32 (27 [26–31]; Figure [C]), resulting in 4 cardiovascular events (sudden cardiac death n=3; heart transplantation n=1; Figure [D]). Patients with HET-GOF variants have a median age of 14 [6–33] and 53% were men (Figure [B]). HET-GOF variants c.1A>G, c.1A>T, c.2T>C, c.3G>T, c.3G>A and c.22A>T (p.[Val2_Met29del]) segregated with disease in 34 families, reporting 62 patients in 14 countries. All patients with HET-GOF had epidermolysis bullosa simplex at birth, and 27% were diagnosed with DCM. The probability of diagnosing DCM during lifetime was significantly different (*P*<0.001, weighted *P<*0.001) between men (25 [16–34], n=9) and women (45 [31–47], n=8; Figure [E]). For all patients with HET-GOF variants, 7 cardiovascular events (sudden cardiac death n=1; heart failure n=4; heart transplantation n=2) were reported. Figure [F] shows the general differences in cardiovascular events between sexes (*P*=0.026, weighted *P=*0.073). The median age of cardiovascular events for men was 20 (n=4) years and 54 years for women (n=3).

**Figure.**
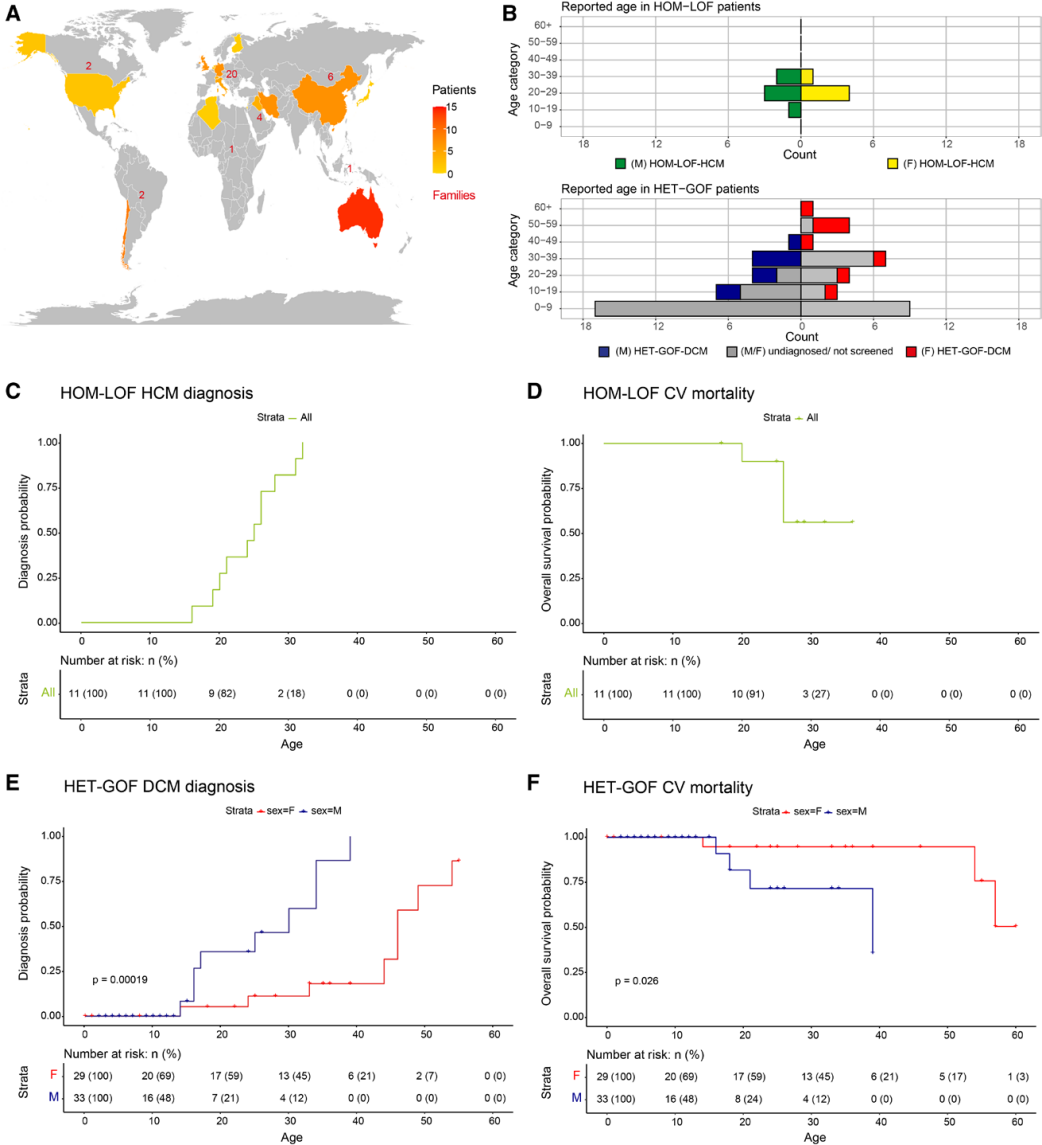
**Severe and early-onset cardiomyopathy in patients with pathogenic *KLHL24* variants. A**, World map color-coding the distribution of patients per country, with the amount of reported families (red) depicted as a number within each continent (North America, South America, Europe, Africa, The Middle East, Asia, and Oceania). **B**, Age and diagnosis distribution of patients by cardiomyopathy diagnosis (hypertrophic cardiomyopathy [HCM], dilated cardiomyopathy [DCM], and undiagnosed/not screened) and stratified by sex. **C**, Kaplan-Meier curve for age of HCM diagnosis of all patients with HOM-LOF. **D**, Kaplan-Meier curve for cardiovascular death of all homozygous loss-of-function (HOM-LOF) patients. **E**, Kaplan-Meier curves, stratified by sex, for age of DCM diagnosis of all patients with heterozygous gain-of-function variants (HET-GOF). **F**, Kaplan-Meier curves, stratified by sex, for cardiovascular death of all patients with heterozygous gain-of-function variants (HET-GOF). F indicates female; and M, male.

This study reports three main findings. First, patients with HOM-LOF variants were diagnosed at an early age with a severe form of HCM, which is strikingly early compared with other, large HCM cohorts. We, therefore, recommend placing all preadolescent carriers with (suspected) HOM-LOF variants under cardiac observation. The low number of patients prevents more extensive analysis. HOM-LOF variants, abolish KLHL24-mediated substrate targeting, resulting in desmin-overload, and subsequently hypertrophy.^4^ Whether heterozygous loss-of-function carriers are also prone to develop HCM remains elusive, as merely 4 carriers with similar genetic/ethnic background have been described. In fact, variants that introduce a premature termination codon before the substrate-recognition-site, or variants that disrupt domains responsible for substrate targeting, could result in LOF. With a pLi score of 0, *KLHL24* is extremely intolerant to LOF. The GenomAD database has reported hundreds of (potential) HET-LOF variants that remain uninvestigated.

Other findings of this study are that men with HET-GOF variants were diagnosed with DCM earlier than women and men also have a higher probability for cardiovascular events at a younger age than women. HET-GOF variants originate in the N-terminus and a hotspot c.1_84 has been identified, where translation initiation (c.1A>G) or translation re-initiation (c.22A>T, premature termination codon) variants cause the same protein pathophysiology. The GenomAD database also reported another HET-GOF variant (c.70C>T, premature termination codon) in humans, that likely result in translation re-initiation. The resulting N-terminal truncation (p.[Val2_Met29del]) renders the KLHL24 protein more stable, causing excessive substrate degradation. Furthermore, sex disparities are common in patients with DCM and typically observed in other genetic cardiomyopathy cohorts. DCM is mostly caused by variants in genes like *TTN* and *LMNA*. Although *TTN* variants are also highly penetrant, most patients develop DCM after the age of 40 years,^5^ which suggests that HET-GOF-*KLHL24* DCM is severe. The number of patients with the same protein pathophysiology (n=62), diverse ethnic background, and segregation among multiple generations, indicates that the correlation of early lethality in men could be strong. We recommend newborns with skin fragility due to HET-GOF variants be kept under clinical observation throughout life, especially when they no longer require dermatological care.

## ARTICLE INFORMATION

### Sources of Funding

This work was supported by the Human Frontier Science Program (grant number RGY 0071/2014 to Dr van der Meer), European Research Counsel (STem Cell Models to Unravel the Susceptibility and Resilience to DevelOP Heart Failure [STOP-HF] starting grant [StG]; grant number 715732, ERC-2016-StG to Dr van der Meer) and in part under the context of the Personalized Medicine in Diabetic Chronic Disease Management (PROMINENT) project (funding from the European Union’s Horizon 2020 research and innovation program under the Marie Skłodowska-Curie grant agreement number 754425).

### Disclosures

Dr Tromp is supported by the National University of Singapore Start-up grant, the tier 1 grant from the Ministry of Education, and the Clinical Scientist-Individual Research Grant (CS-IRG) New Investigator Grant from the National Medical Research Council; has received consulting or speaker fees from Daiichi-Sankyo, Boehringer Ingelheim, Roche diagnostics and Us2.ai, owns patent US-10702247-B2 unrelated to the present work. Dr van der Meer received grant support or consultancy fees from: Novartis, Pharma Nord, Pfizer, Ionis, Astra Zeneca, Vifor Pharma, Pharmacosmos, BridgeBio, NovoNordisk. The other authors report no conflicts.
